# Role of IL-24 in the mucosal remodeling of children with coeliac disease

**DOI:** 10.1186/s12967-020-02221-2

**Published:** 2020-01-23

**Authors:** Réka Rokonay, Apor Veres-Székely, Beáta Szebeni, Domonkos Pap, Rita Lippai, Nóra J. Béres, Gábor Veres, Attila J. Szabó, Ádám Vannay

**Affiliations:** 1 1st Department of Paediatrics, Semmelweis University, 54 Bókay Street, Budapest, 1083 Hungary; 2 MTA-SE Paediatrics and Nephrology Research Group, Hungarian Academy of Sciences and Semmelweis University, Budapest, Hungary; 3 Pediatric Institute-Clinic, University of Debrecen, Debrecen, Hungary

**Keywords:** Coeliac disease, Tissue remodeling, Interleukin 24, Interleukin 20 subfamily, Epithelial cell, Myofibroblast, Peripheral blood mononuclear cells (PBMC)

## Abstract

**Background:**

Recently, involvement of IL-19, IL-20 and IL-24 has been reported in inflammatory diseases associated with tissue remodeling. However, their impact on the pathomechanism of coeliac disease (CD) is still completely unknown.

**Methods:**

Expression of *IL19*, *IL20* and *IL24* was measured by real-time RT-PCR, protein amount of IL-24, α smooth muscle actin (α-SMA) and fibronectin (FN) was determined by Western-blot analysis in the duodenal biopsies of therapy naive children with CD and controls. Localization of IL-24 and IL-20RB was investigated by immunofluorescent staining in the duodenal mucosa. Effect of recombinant IL-1β, TNF-α, TGF-β and IL-17 treatment on the expression of *IL19*, *IL20*, *IL24* and their receptors was investigated by real-time RT-PCR in small intestinal epithelial cells (FHs74Int), in primary duodenal myofibroblasts (pdMFs) and in peripheral blood mononuclear cells (PBMCs). Effect of IL-24 on H_2_O_2_ treated FHs74Int cells and on pdMFs was measured by MTT, LDH, Annexin V assays, real-time RT-PCR and by fluorescent microscopy.

**Results:**

We found increased level of IL-24 (3.3×, p < 0.05), α-SMA (2.4×, p < 0.05) and FN (2.3×, p < 0.05) in the duodenal mucosa and increased expression of *IL19* (3.6×, p < 0.05) and *IL24* (5.2×, p < 0.05) in the PBMCs of children with CD compared to that of controls. IL-1β was a strong inducer of *IL24* expression of FHs74Int cells (9.9×, p < 0.05), pdMFs (552.9×, p < 0.05) or PBMCs (17.2×, p < 0.05), as well. IL-24 treatment reduced the number of apoptotic cells (0.5×, p < 0.05) and decreased the expression of inflammatory factors, including *IL1A, IL6* and *TNF* of H_2_O_2_-treated FHs74Int cells. IL-24 decreased the proliferation (0.6×, p < 0.05) of PDGF-B treated pdMFs. Moreover, IL-24 treatment altered the morphology of pdMFs by influencing the size of the angles between stress fibers and the longitudinal axis of the cells (2.0×, p < 0.05) and the expression of cytoskeletal components, including *ACTA2*, *ACTB*, *VIM*, *SNAI1* and *SNAI2.*

**Conclusion:**

Our results suggest that IL-24 plays a significant role in the maintenance of duodenal mucosal integrity in CD.

## Introduction

Coeliac disease (CD) is a chronic, autoimmune enteropathy caused by exposure to dietary gluten in genetically susceptible individuals [1]. CD is highly prevalent, it affects approximately 1% of the population worldwide [2]. Currently, the therapy of CD is limited to the strict life-long gluten free diet (GFD). However, the high number of patients with non-responsive CD and also the significant therapeutic burden of GFD necessitate the better understanding the pathomechanism of the disease.

The interleukin (IL)-20 subfamily of cytokines is part of the IL-10 family and comprises five related molecules, including IL-19, IL-20, IL-22, IL-24 and IL-26 [3]. The best-known function of the subfamily is to establish a link between leukocytes and epithelial cells, thereby enhancing innate defence mechanism and repairing processes at the epithelial surfaces [4]. These cytokines are mainly produced by immune cells [5, 6], however, other cell types, such as epithelial cells or myofibroblasts (MFs) can also release them [7, 8]. IL-19, IL-20 and IL-24 form a separate group within the subfamily, as they use the common IL-20RA/IL-20RB and IL-22RA/IL-20RB receptor heterodimers [9, 10].

Recently, increased expression of IL-19, IL-20 and IL-24 has been demonstrated in the colonic mucosa and also in peripheral blood mononuclear cells (PBMCs) of patients with inflammatory bowel diseases (IBD) [11–15]. However, to the best of our knowledge, there are no previous data available about the significance of IL-19, IL-20 and IL-24 in CD.

In the present study, we hypothesized that cytokines of the IL-20 subfamily may influence the repair process of the chronically inflamed small intestinal mucosa. Therefore, we examined the expression of IL-19, IL-20 and IL-24 and their receptors in duodenal biopsies of pediatric patients with CD. In vitro experiments were performed to determine the effect of inflammatory cytokines on the expression of the investigated interleukins and their receptors. Finally, as duodenal epithelial cells and duodenal MFs play prominent role in the remodeling of injured mucosa, we examined the effect of IL-24 on these cell types.

## Methods

### Duodenal biopsies

Mucosal biopsies of therapy naive pediatric CD patients (n = 16, age: 6.75 ± 3.38 years) and controls (n = 14, age: 12.71 ± 4.81 years) (Additional file 1) were collected at the 1st Department of Pediatrics, Semmelweis University, Hungary. Written informed consent was obtained from parents of each participant prior to the procedure and the study was approved by the Semmelweis University Regional and Institutional Committee of Science and Research Ethics (TUKEB 58/2013). CD was diagnosed based on the symptoms, serology (anti-tissue transglutaminase IgA and IgG) and duodenal histology (Marsh criteria), according to the criteria of the European Society for Pediatric Gastroenterology, Hepatology, and Nutrition (ESPGHAN) [16]. Control subjects were referred with recurrent abdominal pain, growth retardation and diarrhoea and an upper gastrointestinal endoscopy was part of their diagnostic procedure. Biopsies were immediately snap-frozen and stored at − 80 °C until further analysis.

### Recombinant proteins applied for cell culture experiments

Recombinant human epidermal growth factor (EGF), tumor necrosis factor alpha (TNF-α), interleukin-17 (IL-17), interleukin-24 (IL-24), and platelet-derived growth factor B (PDGF-B) were purchased from R&D Systems (Minneapolis, MN, USA). Recombinant human interleukin-1 beta (IL-1β) and transforming growth factor beta-1 (TGF-β) were purchased from Life Technologies (Carlsbad, CA, USA). EGF, TNF-α, and IL-24 were dissolved in sterile phosphate buffered saline (PBS); IL-1β was dissolved in sterile distilled water; IL-17, TGF-β and PDGF-B were dissolved in 4 mM HCl.

### FHs74Int small intestinal epithelial cell culture

Human small intestinal epithelial cell line (FHs74Int), (American Type Culture Collection (ATCC), Manassas, VA, USA) was cultured in Hybri-Care Medium (ATCC) supplemented with 30 ng/mL EGF, 10% heat-inactivated fetal bovine serum (FBS) (Invitrogen, Carlsbad, CA, USA) and 1% penicillin and streptomycin mixture (Life Technologies) under standard cell culture conditions (37 °C, humidified, 5% CO_2_/95% air environment).

For real time RT-PCRs the cells were seeded into 6 or 96 well plates at a density of 10^5^ or 10^4^ cells/well and treated with IL-1β (100 ng/mL), TNF-α (10 ng/mL), TGF-β (0.5 nM), or IL-17 (100 ng/mL) on 6 well plates (n = 6 well/treatment group), or with IL-24 (0.1 ng/mL) and H_2_O_2_ (1000 µM) on 96 well plates (n = 5 well/treatment group) for 24 h, respectively.

For MTT and LDH assays the cells were seeded into 96-well plates at a density of 10^4^ cells/well (n = 5 well/treatment group) and treated with IL-24 (0.1 ng/mL) and H_2_O_2_ (200, 400, 600, 800 or 1000 µM) (Sigma-Aldrich) for 24 h.

The cells were seeded into 6 well plates at a density of 3 × 10^5^ cells/well (n = 3 well/treatment group) for the Annexin V apoptosis assay, and treated with IL-24 (0.1 ng/mL) and H_2_O_2_ (1000 µM) for 48 h.

Vehicle treated cells served as controls in all experiments.

### Primary duodenal myofibroblast cell culture (pdMFs)

PdMFs were freshly isolated from the duodenal mucosa of control children according to a method described previously [17]. PdMFs were cultured in Dulbecco’s Modified Eagle Medium (Invitrogen) supplemented with 1% FBS and 1% Penicillin and Streptomycin mixture under standard cell culture conditions (37 °C, humidified, 5% CO_2_/95% air environment).

For real time RT-PCRs the pdMFs were seeded into 6 or 96 well plates at a density of 10^5^ or 10^4^ cells/well and treated with IL-1β (100 ng/mL), TNF-α (10 ng/mL), TGF-β (0.5 nM), or IL-17 (100 ng/mL) on 6 well plates (n = 6 well/treatment group), or with IL-24 (0.01 ng/mL) and PDGF-B (10 ng/mL) on 96 well plates (n = 5 well/treatment group) for 24 h, respectively.

To investigate the orientation of α-SMA immunopositive stress fibers, pdMFs were seeded in 4-well cell culture chambers at a density of 2 × 10^4^ cells/well and treated with TGF-β (0.5 nM) or IL-24 (0.1 ng/mL) only, or co-treated with IL-24 (0.1 ng/mL) and TGF-β (0.5 nM) for 24 h. For MTT and LDH assays pdMFs were seeded into 96-well plates at a density of 10^4^ cells/well (n = 5 well/treatment group) and treated with IL-24 (0.01, 0.1, 1, 10 or 100 ng/mL) and PDGF-B (10 ng/mL) for 24 h.

To perform Sirius Red assay, pdMFs were seeded into 96-well plates at a density of 10^4^ cells/well (n = 5 well/treatment group), and treated with 0.01, 0.1, 1, 10 or 100 ng/mL IL-24 and 0.5 nM TGF-β for 48 h.

Vehicle treated cells served as controls in all experiments.

### Peripheral blood mononuclear cells (PBMCs)

In this experiment, 8 children with CD on gluten-free diet (age: 10.86 ± 2.99 years) and 8 controls (age: 10.30 ± 4.62 years) were enrolled. Control subjects had gastrointestinal symptoms, but during their gastroenterological examination CD was excluded. PBMCs were isolated by density gradient centrifugation using Histopaque-1077 (Sigma-Aldrich). After isolation, the cells were placed into RPMI 1640 medium (ATCC) supplemented with 10% FBS and 1% penicillin and streptomycin mixture.

For real time RT-PCRs, PBMCs derived from CD patient were seeded into 24-well plates at a density of 5 × 10^5^ cells/well (n = 6 well/treatment group) and treated either with IL-1β (100 ng/mL), TNF-α (10 ng/mL), TGF-β (0.5 nM) or IL-17 (100 ng/mL) for 24 h.

Vehicle treated cells served as controls.

### Immunohistochemistry

Frozen duodenal biopsy samples derived from controls and children with CD were embedded into Shandon cryomatrix (ThermoElectron Co., Madison, WI, USA) and cut into 5 μm sections. Samples were incubated with primary antibodies specific for human IL-24 (ab182567; rabbit, 1:100, Abcam, Cambridge, United Kingdom) or IL-20RB (ab124332; rabbit, 1:100, Abcam) for 1 h at room temperature (RT). After repeated washing, slides were incubated with the corresponding Alexa Fluor 568 secondary antibody (1:200 anti-rabbit, Invitrogen) for 30 min at RT in the dark and counterstained with Hoechst 33342 (1:2000, Sigma-Aldrich). Finally, sections were coverslipped with Vectashield fluorescent mounting medium (Vector Laboratories, Burlingame, Calif., USA). Appropriate controls were performed by omitting the primary antibody to assure specificity and to avoid autofluorescence. Sections were analyzed with a Nikon C2 confocal laser scanning microscope system (Nikon, Minato, Tokyo, Japan).

### Immunocytochemistry

FHs74Int and MFs were seeded in chambers and cultured for 24 h in 37 °C. After repeated washing slides were permeabilized with Cytofix/Cytoperm (BD Pharmingen, San Diego, CA, USA) for 15 min at RT, then washed again, and incubated with primary antibody specific for IL-20RB (ab124332; rabbit, 1:100, Abcam) or α-SMA (sc-53015; mouse, 1:1000, Santa Cruz Biotechnology, Dallas, TX, USA) for 1 h at RT. Thereafter the slides were washed with WashPerm solution and incubated with the corresponding Alexa Fluor 568 or 488 conjugated secondary antibody (1:200 anti-rabbit or anti-mouse, Invitrogen) for 30 min (min) at RT in the dark and counterstained with Hoechst 33342 (1:2000, Sigma-Aldrich). Finally, slides were coverslipped with Vectashield fluorescent mounting medium (Vector Laboratories). Appropriate controls were performed by omitting the primary antibodies to assure their specificity and to avoid autofluorescence. Sections were analyzed with a Nikon C2 confocal laser scanning microscope system (Nikon Corporation, Tokyo, Japan).

### RNA isolation, reverse transcription and real-time RT-PCR

Total RNA was isolated from duodenal biopsy samples, FHs74Int cells, pdMFs or PBMCs by Geneaid Total RNA Mini Kit (Geneaid Biotech Ltd., New Taipei City, Taiwan). RNA (500 ng/sample) was reverse-transcribed using SuperScript III reverse transcriptase (Life Technologies) to generate first-stranded cDNA. The mRNA expression of the investigated genes was determined by real-time PCR using LightCycler 480 SYBR Green I Master enzyme mix on a Light Cycler 480 system (Roche Diagnostics, Mannheim, Germany). The nucleotide sequences and annealing temperatures of the primer pairs and the resulted PCR product lengths are shown in Additional file 2 [18]. Relative mRNA expression was determined by comparison with *RPLP0* as internal control using the ∆∆Ct method [19]. Data were normalized and presented as the ratio of their control values.

### Protein isolation and Western blot analysis

Duodenal biopsy samples were homogenized in lysis buffer, containing 50 mM HEPES, 150 mM NaCl, 1% Triton X-100, 5 mM EDTA, 5 mM EGTA, 20 mM sodium pyrophosphate, 20 mM NaF, 0.2 mg/mL phenylmethylsulfonyl fluoride, 0.01 mg/mL leupeptin, and 0.01 mg/mL aprotinin (pH 7.4) (each substance was obtained from Sigma-Aldrich). Thereafter protein concentration was determined by a detergent-compatible protein assay (Bio-Rad, Hercules, CA). Denatured samples [20] (20 μg protein/lane) were separated on 4-20% gradient SDS polyacrylamide gel, and transferred to nitrocellulose membranes. The nitrocellulose membranes were blocked with 5% non-fat milk in tris-buffered saline (TBS) for 1 h at RT. Thereafter, they were incubated overnight at 4 °C with antibodies specific for human IL-24 (ab182567; 1:1000, Abcam), α-SMA (sc-53015; 1:10,000, Santa Cruz Biotechnology), FN (ab2413; 1:2000, Abcam) or GAPDH (sc-47724; 1:2000, Santa Cruz Biotechnology). After repeated washing with TBS containing 0.05% Tween-20 and 1% non-fat milk, membranes were incubated with the corresponding horseradish peroxidase-conjugated secondary antibodies (1:2000 anti-rabbit or anti-mouse, Santa Cruz Biotechnology) for 1 h at RT. Bands of interest were detected using enhanced chemiluminescence detection (Western Blotting Luminol Reagent, GE Healthcare, Waukesha, WI) and quantified by densitometry (VersaDoc, Quantity One Analysis software; Bio-Rad) as integrated optical density after background subtraction. Relative protein levels were determined by comparison with GAPDH as internal control. Data were normalized and presented as the ratio of control values.

### MTT cell proliferation and viability assay

MTT cell proliferation and viability assay was performed by using Cell Proliferation Kit I (Roche Diagnostics) according to the manufacturer’s recommendations. Absorbance was recorded at 570 nm and at 690 nm as background using a Hidex Chameleon Microplate Reader (Triathler, Plate Chameleion, 300SL Lablogic Systems, Inc., Brandon, FL, USA) using MikroWin 2000 software.

### LDH cytotoxicity assay

LDH assay was performed as previously described [21]. All reagents were purchased from Sigma-Aldrich. Absorbance was recorded at 570 nm and at 690 nm as background in a Hidex Chameleon Microplate Reader using MikroWin 2000 software.

### Apoptosis detection assay

The apoptosis assay was performed using FITC Annexin V Apoptosis Detection Kit I (BD Pharmingen) according to the manufacturer’s recommendations. Cells that are negative for Annexin V and PI are refered as viable cells, cells that are positive for Annexin V and negative for PI are refered as early apoptotic cells and cells that are positive for Annexin V and PI are referec as late apoptotic cells. Necrotic cell debris are negative for Annexin V and positive for PI. The flow cytometry analysis was performed using a FACS Aria cytometer (BD).

### SiriusRed collagen detection assay

The SiriusRed assay was performed as previously described [22]. All reagents were purchased from Sigma-Aldrich. Absorbance was determined at 544 nm and at 690 nm as background using Hidex Chameleon Microplate Reader using MikroWin 2000 software.

### Graphical analysis of stress fiber orientation

Assessment of the orientation of α-SMA immunopositive stress fibers in pdMFs was carried out by graphical analysis. Images of 35–40 randomly selected cells from each treatment group were taken using the Olympus IX81 fluorescent microscope system (Olympus Corporation, Tokyo, Japan). To properly represent the whole-cell stress fiber network, the angle between at least 20 stress fibers per cell and the long axis of MFs were determined by ImageJ 1.48v software (The National Institutes of Health, Bethesda, MD) [23]. The parallelism of stress fibers with the long axis was defined as the width of 95% confidence interval of the measured angels in each cell.

### Statistical analysis

Statistical evaluation of data was performed by GraphPad Prism 6.01 software (GraphPad Software Inc., La Jolla, CA, USA). After testing normality with the Kolmogorov–Smirnov test, raw data of real-time RT-PCR, Western blot and stress fiber orientation measurements were analyzed with Mann–Whitney U-test or Kruskal–Wallis test to determine the differences between corresponding groups. Multiple comparisons of the raw data derived from MTT, LDH, Annexin V apoptosis and SiriusRed assays were performed by using multiple t-test and ordinary two-way ANOVA with Dunnett correction. p ≤ 0.05 was considered as statistically significant. Values were expressed as mean + SD.

## Results

### Alteration of IL-19, -20 and -24, IL-20RB, α-SMA and FN in the duodenal mucosa of children with CD

**Fig. 1:**
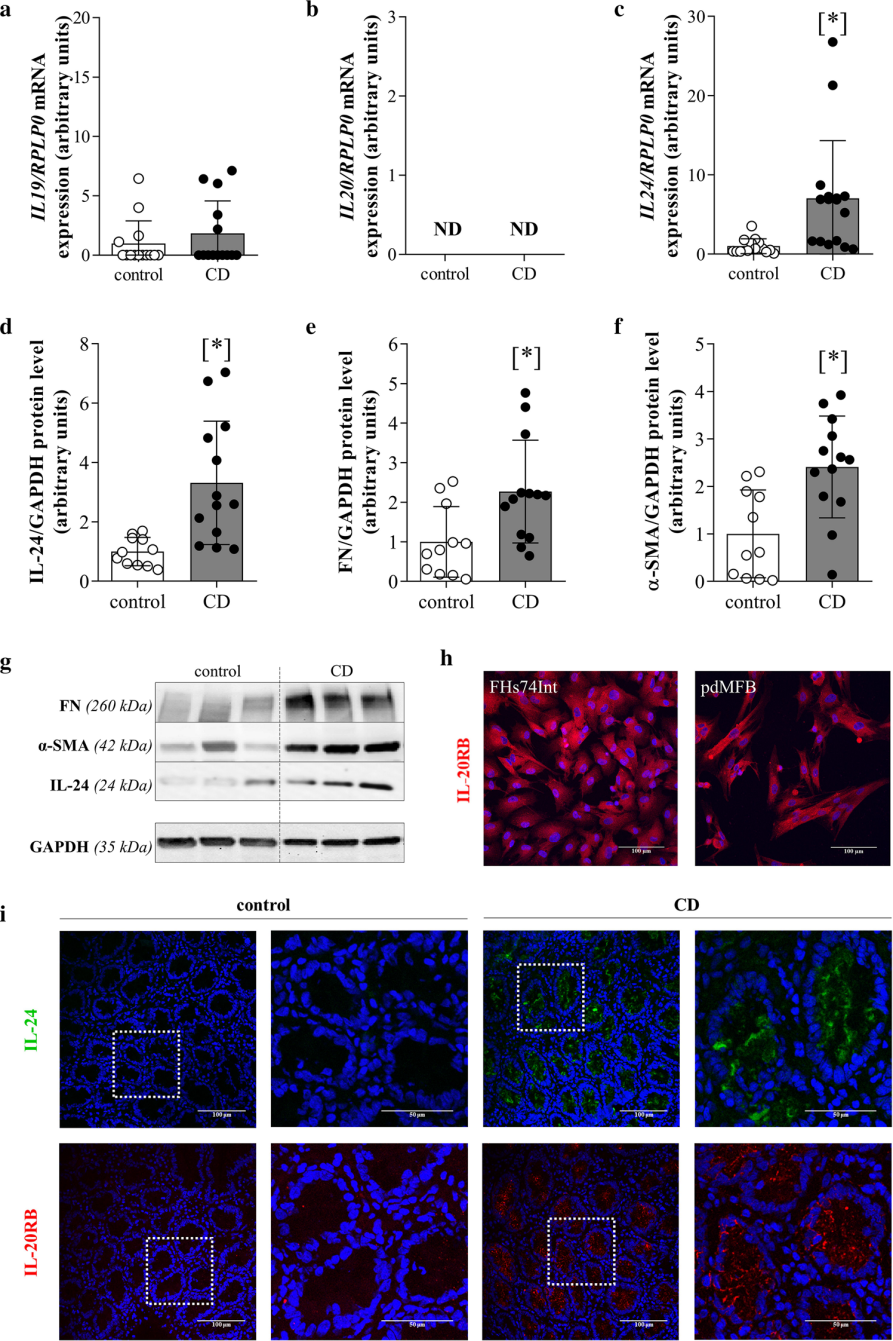
Alteration of IL-19, -20 and -24, IL-20RB, α-SMA and FN in the duodenal mucosa of children with CD. The mRNA expression of *IL19* (**a**), *IL20* (**b**) and *IL24* (**c**) of the duodenal mucosa of children with CD (n = 16) and controls (n = 14) was determined by real-time RT-PCR in comparison with *RPLP0* as internal control. Relative protein amounts (and representative immunoblots) of IL-24 (**d**, **g**), FN (**e**, **g**) and α-SMA (**f**, **g**) of the duodenal mucosa of children with CD (n = 13) and controls (n = 11) were investigated by Western blot analysis in comparison with GAPDH as internal control (**g**). Results are presented as mean ± SD. *p < 0.05 vs. control (Mann–Whitney U-test). Presence of IL-20RB (red) in FHs74Int cells and pdMFs (**h**) and the localisation of IL-24 (green) and IL-20RB (red) in the duodenal mucosa of children with CD and controls (**i**) was determined by immunofluorescence staining. Cell nuclei were counterstained with Hoechst 33342 (blue). Magnified regions are highlighted by white squares. Scale bar: 100 µm and 50 µm

The mRNA expression of *IL24* was increased in the duodenal mucosa of children with CD compared to that of controls (Fig. 1c). There was no difference in the mRNA expression of *IL19* (Fig. 1a) between the groups, and the expression of *IL20* (Fig. 1b) was undetectable. Protein amounts of IL-24, FN and α-SMA were elevated in the duodenal mucosa of children with CD compared to controls (Fig. 1d–f). Strong IL-24 and IL-20RB immunoreactivity was observed in the duodenal crypt enterocytes of children with CD (Fig. 1i). IL-20RB immunopositivity was observed on FHs74Int intestinal epithelial cells and on pdMFs, as well (Fig. 1h).

### Effect of IL-1β, TNF-α, TGF-β and IL-17 treatment on the expression of *IL*-*19,* -*20,* -*24* and their receptors

**Fig. 2:**
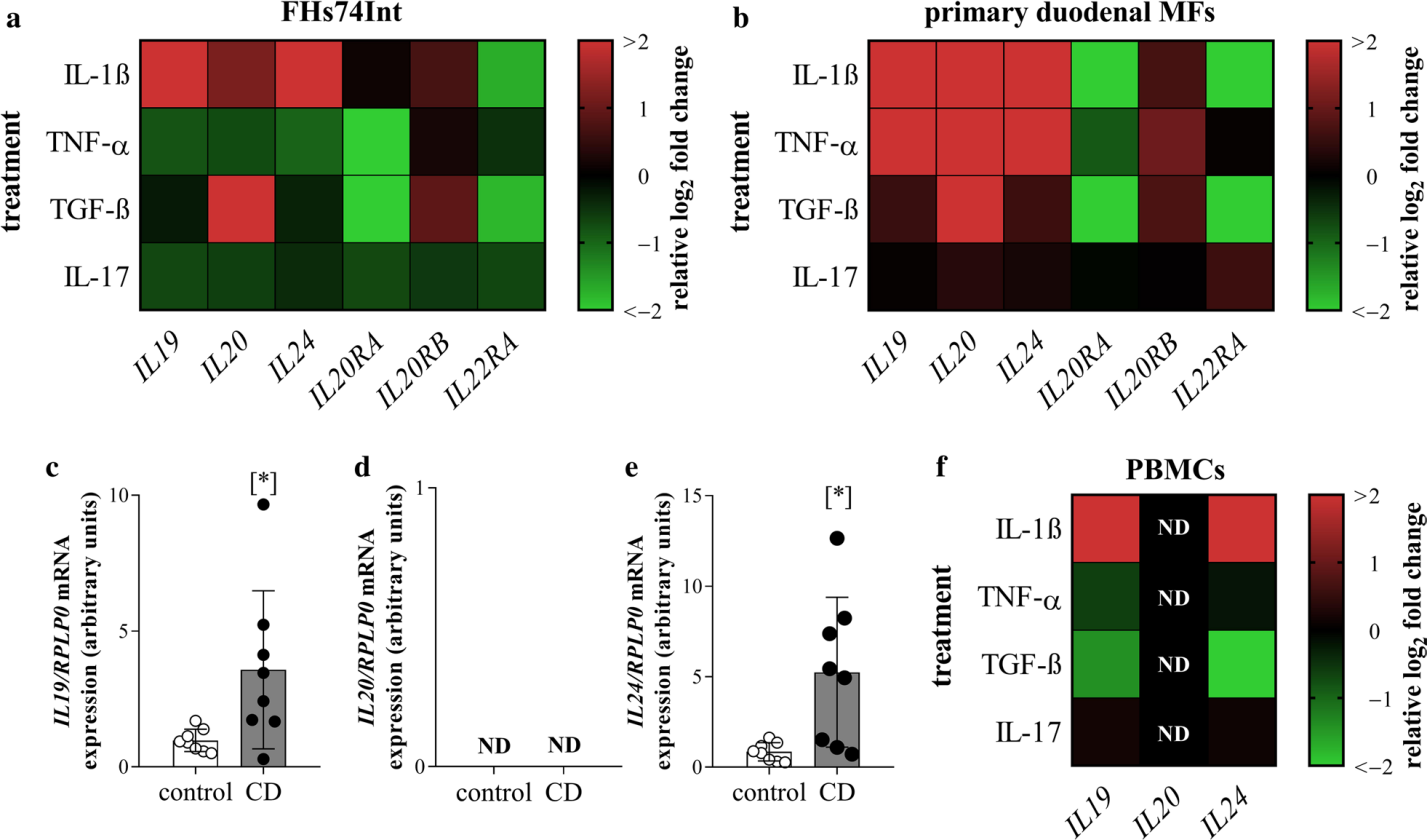
Effect of IL-1β, TNF-α, TGF-β and IL-17 treatment on the expression of *IL19, IL20, IL24* and their receptors. The mRNA expression of *IL19*, *IL20*, *IL24, IL20RA*, *IL20RB* and *IL22RA* was determined by real-time RT-PCR in comparison with *RPLP0* as internal control. Heat maps illustrate the mean fold change in relative expression of the examined genes after different treatments in FHs74Int cells (**a**), pdMFs (**b**) and PBMCs of children with CD (**f**) compared to vehicle controls (n = 6). Graphs of individual measurements can be found in Additional files 3, 4, 5, 6, 7. The mRNA expression of *IL19* (**c**), *IL20* (**d**) and *IL24* (**e**) was determined in the PBMCs of 8–8 children with CD and controls, respectively. Results are presented as mean ± SD. ND: not detectable; *p < 0.05 vs. control (Mann–Whitney U-test)

In FHs74Int cells IL-1β treatment increased, TNF-α and IL-17 treatment decreased the mRNA expression of *IL19*, *IL20* and *IL24* (Fig. 2a, Additional file 3). TGF-β treatment increased the mRNA expression of *IL20*. TGF-β and TNF-α treatment decreased the mRNA expression of *IL20RA*. IL-1β or TGF-β treatment increased the mRNA expression of *IL20RB* and decreased that of *IL22RA*. IL-17 treatment decreased the expression of *IL22RA* in FHs74Int cells (Fig. 2a, Additional file 4).

In pdMFs IL-1β and TNF-α treatment increased the mRNA expression of *IL19*, *IL20* and *IL24,* and TGF-β treatment increased the mRNA expression of *IL20* (Fig. 2b, Additional file 5). IL-1β, TNF-α, and TGF-β treatment decreased the expression of *IL20RA* and increased that of *IL20RB*. The mRNA expression of *IL22RA* was increased by IL-17 and decreased by IL-1β and TGF-β treatment (Fig. 2b, Additional file 6).

Comparing their *IL19*, *IL20* and *IL24* mRNA expressions, significantly higher *IL19* and *IL24* expression was observed in the PBMCs of children with CD compared to controls (Fig. 2c–e). In PBMCs derived from patient with CD, IL-1β treatment increased, and TGF-β treatment decreased the mRNA expression of *IL19* and *IL24*. *IL20* mRNA was not detectable either in the untreated or treated groups (Fig. 2f, Additional file 7).

### Effect of IL-24 treatment on the viability of H_2_O_2_ treated FHs74Int cells

**Fig. 3:**
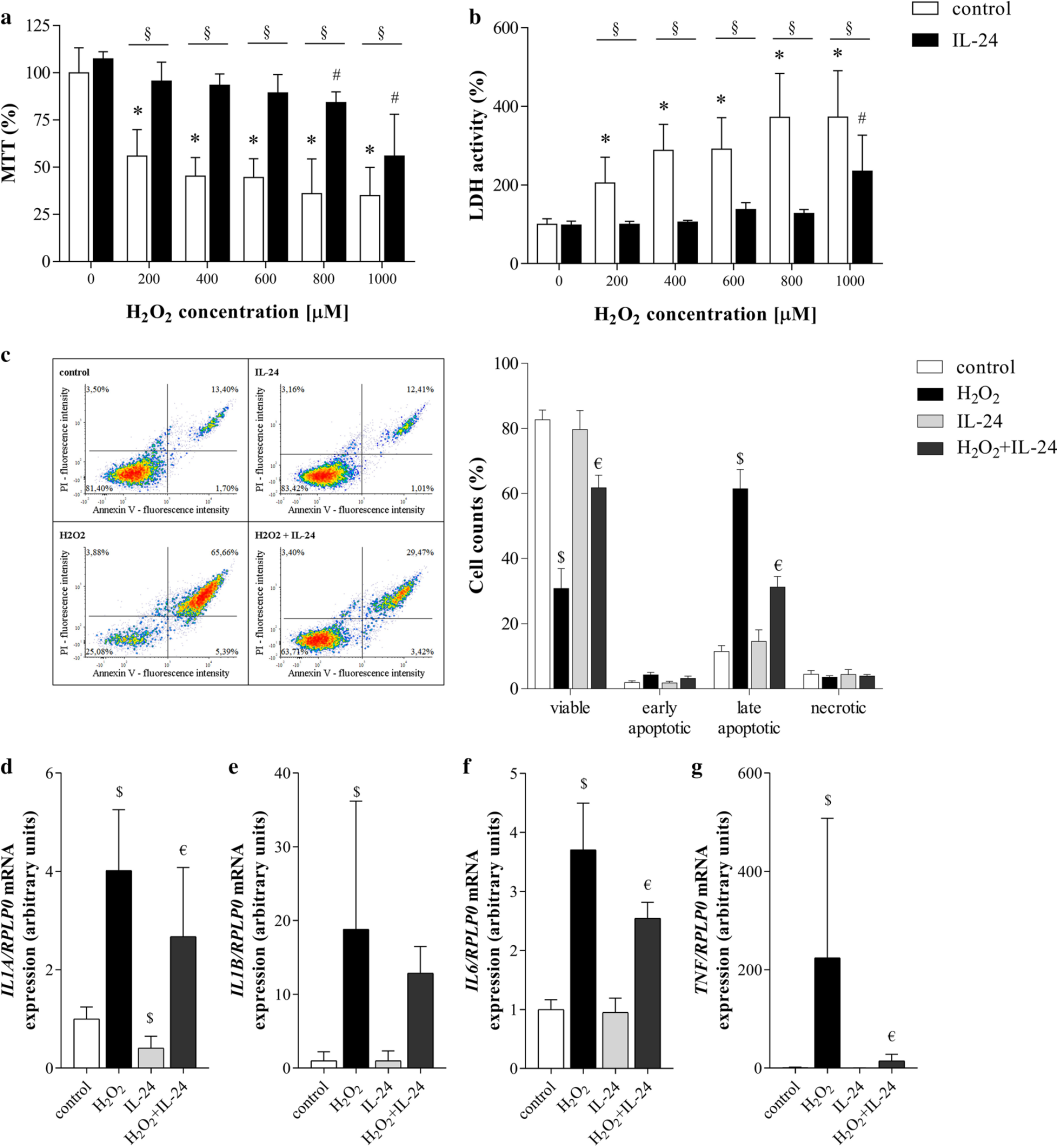
Effect of IL-24 treatment on the viability of H_2_O_2_ treated FHs74Int cells. The effect of IL-24 on the viability of the H_2_O_2_ treated cells was investigated by MTT (**a**), LDH (**b**) and Annexin V apoptosis (**c**) assays. Results of MTT and LDH assays (n = 5) are presented as the percentage of the untreated group (0 µM H_2_O_2_ control), and as the percentage of total cells in the case of Annexin V apoptosis assay (n = 3 × 3 replicates). The mRNA expression of *IL1A* (**d**), *IL1B* (**e**), *IL6* (**f**) and *TNF* (**g**) was determined by real-time RT-PCR in comparison with *RPLP0* as internal control (n = 5). Results are presented as mean + SD. *p < 0.05 vs. 0 µM *H*_*2*_*O*_*2*_*control* (two-way ANOVA); ^#^p < 0.05 vs. *IL*-*24 *+0 µM *H*_*2*_*O*_*2*_ (two-way ANOVA); §p < 0.05 *control* vs. *IL*-*24* at the concerning H_2_O_2_ concentration (multiple t-test); $p < 0.05 vs. control (two-way ANOVA); ^€^p < 0.05 vs. H_2_O_2_ (two-way ANOVA)

Decreased viability of H_2_O_2_ treated FHs74Int cells was improved by IL-24 (Fig. 3a). Accordingly, LDH activity in the supernatant of H_2_O_2_ treated cells was significantly reduced by IL-24 (Fig. 3b), and IL-24 treatment reduced the number of apoptotic (Annexin V and PI positive) cells in the H_2_O_2_ treated group from 61% to 31% (Fig. 3c). IL-24 treatment decreased the H_2_O_2_ induced mRNA expression of *IL1A, IL6* and *TNF*, as well (Fig. 3d, f, g).

### Effect of IL-24 treatment on the proliferation of pdMFs

**Fig. 4:**
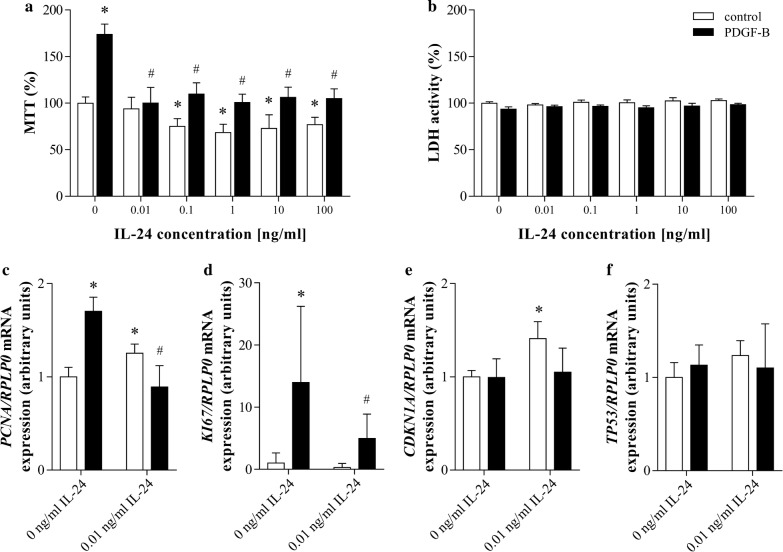
Effect of IL-24 treatment on the proliferation of pdMFs. Cell proliferation and cytotoxicity was investigated by MTT (**a**) and LDH (**b**) assays (n = 5). Results are presented as the percentage of the untreated group (0 ng/mL IL-24 control). The mRNA expression of *PCNA* (**c**), *KI67* (**d**), *CDKN1A* (**e**), and *TP53* (**f**) was determined by real-time RT-PCR (n = 5) in comparison with *RPLP0* as internal control. Results are presented as mean + SD *p < 0.05 vs. 0 ng/mL *IL*-*24 control* (two-way ANOVA); ^#^p < 0.05 vs. *PDGF*-*B *+0 ng/mL *IL*-*24* (two-way ANOVA)

We found that IL-24 treatment decreased the endogenous and also the PDGF-B induced proliferation of pdMFs as suggested by the MTT assay (Fig. 4a). However IL-24 treatment did not affect the LDH release of the cells demonstrating that it did not affect the viability of PDGF-B treated pdMFs (Fig. 4b). Accordingly, IL-24 treatment decreased the PDGF-B induced expression of proliferation markers, including *PCNA* and of *KI67*, but had no effect on the expression of the regulators of apoptosis including *CDKN1A* or *TP53* (Fig. 4c–f).

### Effect of IL-24 treatment on the stress fiber orientation of pdMFs

**Fig. 5:**
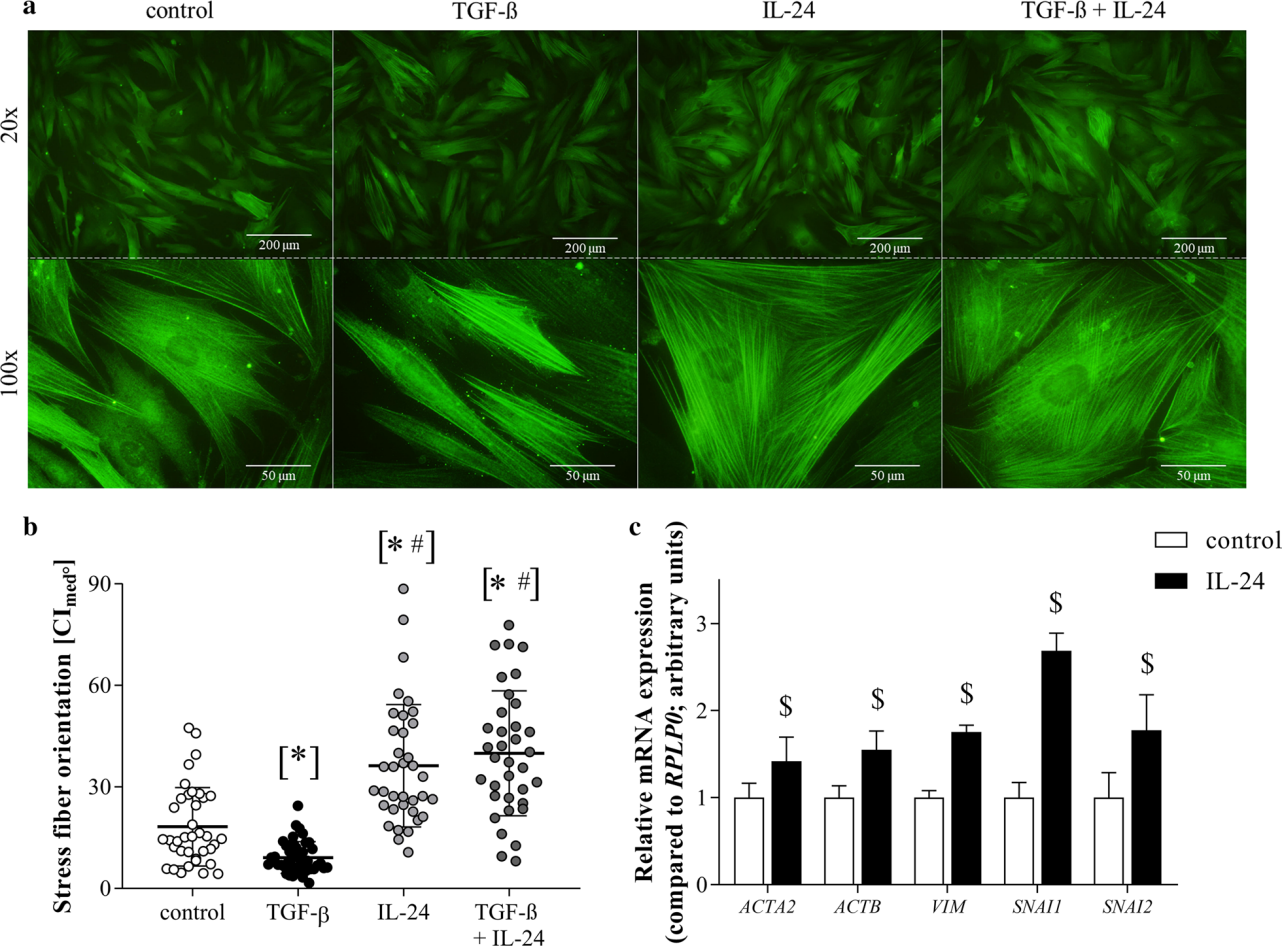
Effect of IL-24 treatment on the stress fiber orientation of pdMFs. α-SMA immunopositive stress fibers was visualized by immunofluorescence staining (green) (**a**). The orientation of stress fibers was assessed by graphical analysis (**b**) using ImageJ 1.48v software. Plots represent the width of 95% confidence intervals of the measured angels between stress fibers and the long axis of the investigated cell (n = 35–40). Horizontal lines indicate mean ± SD. Scale bar: 200 µm or 50 µm. Relative mRNA expression of *ACTA2*, *ACTB*, *VIM*, *SNAI1* and *SNAI2* (**c**) was determined by real-time RT-PCR (n = 5) in comparison with *RPLP0* as internal control. Results are presented as mean + SD. *p < 0.05 vs. control (Kruskal–Wallis test), ^#^p < 0.05 vs. TGF-β (Kruskal–Wallis test). ^$^p < 0.05 vs. control (Mann–Whitney U-test)

Elongated morphology with highly parallel α-SMA immunopositive stress fibers were observed in untreated and TGF-β treated pdMFs. Orientation of the stress fiber system and cell morphology of pdMFs was significantly changed by IL-24, as the angles between the stress fibers and the longitudinal axis of the cells increased, and circular fiber displacement and sheet-like cell shape were experienced after IL-24 treatment (Fig. 5a, b). Accordingly, IL-24 treatment increased the mRNA expression of *ACTA2*, *ACTB*, *VIM*, *SNAI1* and *SNAI2* as it was compared to the controls (Fig. 5c).

## Discussion

The architecture of the small intestinal mucosa is maintained by a precisely controlled balance among enterocyte proliferation in the crypts, migration along the villi and shedding of the senescent epithelial cells at the tip of the villi into the intestinal lumen [24]. This fine-tuned process requires tight cooperation between enterocytes and the underlying subepithelial MFs [25]. In CD the chronic mucosal inflammation disturbs this fine-tuned process leading to villous atrophy and crypt hyperplasia.

In the present study we investigated the role of IL-19, IL-20 and IL-24 in mucosal remodeling in CD, which is indicated by the increased amount of MF marker α smooth muscle actin (α-SMA) and the extracellular matrix (ECM) component fibronectin (FN) in the inflamed duodenal mucosa of children with CD (Fig. 1e–g). Recently, the role of IL-20 cytokine subfamily has been proposed in tissue remodeling [26], and increased expression of IL-19, -20 and -24 was demonstrated in PBMCs and also in colonic mucosa of adult IBD patients [11, 13–15]. It has been suggested that these cytokines may protect the integrity of the inflamed mucosa of patients with IBD. However, to the best of our knowledge our study is the first one that investigated the role of the subfamily in the pathomechanism of CD.

Although the amount of IL-24 of the investigated children showed no correlation with the clinical parameters (data not shown) we found increased mRNA expression and protein level of IL-24 in the duodenal mucosa of children with CD (Fig. 1c, d) IL-24 was present in the mucosal epithelial layer and also in the lamina propria of CD patients (Fig. 1i). Increased expression of *IL19* and *IL24* was observed in PBMCs isolated from children with CD compared to those of healthy children, as well (Fig. 2c, e). Despite the common features of the investigated cytokines of the IL-20 subfamily, *IL20* was not even detectable in the duodenal mucosa or in PBMCs of children with CD (Figs. 1b, 2d).

We also investigated the role of CD associated inflammatory cytokines and growth factors IL-1β, TNF-α, TGF-β and IL-17 on the expression of IL-19, -20 and -24 in FHs74Int duodenal epithelial cells (Fig. 2a; Additional file 3), pdMFs (Fig. 2b; Additional file 5) and in PBMCs derived from pediatric patient with CD (Fig. 2f; Additional file 7). Although, all factors influenced the production of the investigated cytokines, the effect of IL-1β was the most significant. Indeed, IL-1β treatment increased the expression of all three cytokines in each cell type, except *IL20* expression in PBMCs, which remained undetectable. Our experiments may also explain the increased amount of IL-24 in the duodenal mucosa of children with CD, as the increase in *IL24* expression after IL-1β stimulation was an order of magnitude higher in pdMFs than that of *IL19* or *IL20* (Additional file 5). Our results are in accordance with the observation of Andoh et al. [11], who suggested that IL-1β is the main inducer of IL-24 in subepithelial colon MFs. In contrast, they found that IL-1β treatment has no effect on the *IL24* expression in HT-29, Caco-2 or SW480 colon epithelial cells. However, this contradiction may be due to the different origin of the investigated cell lines. While FHs74Int cells originate from healthy small intestine, the cell lines investigated by Andoh et al. are derived from different colorectal carcinomas.

However, in contrast to Andoh et al. we demonstrated that beside IL-1β other factors may also significantly influence the expression of *IL19*, *IL20* or *IL24*. Indeed, we found that TNF-α also affect the expression of the investigated cytokines but in a cell type dependent manner. While TNF-α treatment increased the expression of *IL19*, *IL20* and *IL24* in the pdMFs it decreased their expression in the FHs74Int epithelial cells and had no effect in the PBMCs (Fig. 2). Previously, it has been described, that TNF-α has cell-type-specific effect on the regulation of genes involved in inflammation, cell homeostasis, proliferation or viability [27]. Lee A. et al. revealed that TNF-α treatment induce a remarkable and sustained IL-6 production of synovial fibroblasts, but not in macrophages, where only a transient increase of IL-6 production was observed. They demonstrated that TNF-α treatment increase the level of the well-known inhibitors of cytokine expression, including ABIN3, IRAK-M, SOCS3 and ATF3 in the macrophages thus inhibiting the expression of IL-6. These factors are present not only in immune cells [28] but also in epithelial cells [29], therefore they may influence the IL-24 expression of the FHs74Int epithelial cells, as well.

Taken together, our results demonstrated that, epithelial cells, MFs and PBMCs are responsible for IL-19, -20 and -24 production in response to mucosal inflammation in CD.

Biological effects of IL-19, -20 and -24 are mediated by IL-20RA/IL-20RB or IL-22RA/IL-20RB receptor complexes. We found that presence of the common IL-20RB subunit was increased on epithelial cells and on lamina propria cells of the duodenal mucosa of children with CD (Fig. 1i) and it was also expressed by FHs74Int cells and pdMFs (Fig. 1h). Our experiments also demonstrated that the expression of *IL20RA*, *IL20RB* and *IL22RA* receptor subunits is tightly regulated by the investigated inflammatory cytokines. Indeed, while most treatment, including IL-1β, TNF-α or TGF-β, increased the synthesis of the *IL20RB* receptor subunit, the same treatments decreased the expression of *IL20RA* or *IL22RA* subunits in FHs74Int cells (Fig. 2a; Additional file 4) and pdMFs (Fig. 2b; Additional file 6). The decreased expression of *IL20RA* or *IL22RA* subunits might inhibit the formation of mature receptor complexes in the inflamed duodenal mucosa. However, it has recently been reported that IL-24 can bind to IL-20RB monomers as well [10]. This interaction then promotes the dimerization of IL-20RB with IL-20RA and IL-22RA subunits, which further enhances the binding affinity of the receptor complexes to their ligands [30]. These data together with the increased mucosal expression of IL-24 suggest its unique importance amongst the investigated cytokines in the pathomechanism of CD.

Thus, the following in vitro experiments we focused on IL-24 to reveal its possible biological role in the pathomechanism of CD. The gluten exposure and the evolving chronic inflammation in CD lead to the overproduction of reactive oxygen species contributing to small intestinal epithelial damage [31]. Investigating the effect of IL-24 on oxidative stress related damage, we found that it exerts a protective effect in epithelial cells according to the results of our MTT, LDH or Annexin V assays (Fig. 3a–c). IL-24 treatment also decreased the expression of *IL1A*, *IL6* and *TNF* inflammatory cytokines in FHs74Int cells (Fig. 3d, f, g). This observation is in accordance with the literature demonstrating the anti-inflammatory effect of the IL-20 subfamily members and also of the larger, IL-10 family of cytokines [4]. Taken together, our results suggest that IL-24 has beneficial effects on the preservation of epithelial layer integrity in the inflamed duodenal mucosa.

The physiological or damage associated renewal of the intestinal epithelial layer highly depends on the integrity of the underlying basement membrane composed of ECM proteins, like collagens, laminin and fibronectin. Components of the basement membrane are produced by subepithelial MFs of the small intestine [32, 33], therefore they play a key role in the regeneration of the coeliac mucosa.

Examining the role of IL-24 on pdMFs we experienced that IL-24 significantly influences their morphology. Whilst in untreated or TGF-β stimulated pdMFs the stress fibers, laid parallel with the longitudinal axis of the cells, we found circular fiber displacement and sheet-like shape of IL-24 treated pdMFs (Fig. 5a, b). Accordingly, IL-24 increased the expression of cytoskeletal structural components (α-SMA (encoded by *ACTA2* gene) and ß-actin (*ACTB*), vimentin (*VIM*) and cell morphology regulators (Snail (*SNAI1*) and Slug (*SCNAI2*)) (Fig. 5c), confirming its regulatory role on stress fiber organization.

Recently, Roncoroni et al. demonstrated that similarly to our IL-24 treated pdMFs, MFs isolated from CD patients are characterized by circular stress fiber orientation and sheet-like shape. These MFs also had decreased motility compared to elongated MFs isolated from controls [24]. They suggested that decreased motility of sheet-like MFs may contributes to disease progression, as the reconstitution of normal epithelial structure may require the migration of MFs to the site of the injury. However, the connection between the orientation of stress fibers and disease progression may be more complex.

Previously, it has been demonstrated that actin-myosin filaments are the major determinant of traction force (TF) generated by the MFs and that the incorporation of α-SMA into the stress fibers significantly enhances the extent of TF [32–34]. It has been also shown that the generated TF is far greater than needed for cell migration, it is rather responsible for the contraction of the ECM by the MFs [35–37]. Moreover, subepithelial MFs are continuously present in healthy and inflamed duodenal mucosa, as well [38]. Therefore, we suggest that the observed effects of IL-24 on the expression of α-SMA and cell morphology may rather contribute to the generation of increased TF and contraction of the surrounding ECM [24].

The effect of IL-24 on proliferation rate and ECM production of pdMFs was also investigated. IL-24 treatment significantly decreased endogenous and also the PDGF-B induced proliferation of pdMFs (Fig. 4a) without affecting their viability (Fig. 4b). Accordingly, we found altered expression of cell cycle regulators in IL-24 treated cells (*PCNA*, *KI67*, *CDKN1A*) (Fig. 4c–f) [39, 40]. Our findings are in accordance with the study of *Liang* et al., demonstrating that IL-24 is a potent anti-proliferative agent, as overexpression of IL-24 inhibits the proliferation of keloid fibroblasts [41]. The expression of *COL1A1*, which encodes collagen type I, the one of the main connective tissue component was slightly increased by IL-24 treatment, but it did not affect the overall ECM production of pdMFs (Additional file 8). Although MFs and ECM components play an essential role in tissue regeneration, their excessive proliferation and ECM production leads to fibrosis and the destruction of the normal tissue architecture [26]. The observed effects of IL-24 on the proliferation and ECM production of pdMFs suggest its role in regenerative tissue remodeling contributing to the reconstitution of normal mucosal architecture in CD.

## Conclusion

In summary although there are limitations of our study, including the number and origin of the human samples, which do not let us to compare the expression of the investigated cytokines between therapy naive children with CD and children with CD receiving GFD, we made great progress in the understanding of the biological effects of IL-24 in the pathomechanism of CD. We demonstrated increased expression of IL-24 in the duodenal mucosa of therapy naive children with CD. We described the regulatory role of IL-1ß, TNF-α, TGF-β and IL-17 on the IL-24 production of FHs74Int duodenal epithelial cells, pdMFs and PBMCs. We showed that IL-24 can protect epithelial cells against oxidative damage, thus it may facilitate the maintenance of epithelial integrity. IL-24 inhibited the proliferation of pdMFs and altered their stress fiber organization, thereby suggesting its role in the reconstitution of normal epithelial structure and mucosal integrity. Currently, the only available therapy of CD is the GFD, which requires increased attention and makes the everyday life difficult, decreasing the compliance of the patients [42]. Moreover, high number of the patients does not respond to the GFD. These unsolved difficulties necessitate the better understanding the pathomechanism of the disease. We hope that our and other work suggesting the importance of inflammatory factors, including IL-15, IL-21, IL-24, or IFN-γ [43], may finally contribute to the identification of novel biomarkers and new drugs to treat CD.

## Supplementary information

**Additional file 1.** Clinical characteristics and laboratory parameters of controls and pediatric CD patients involved into duodenal sample collection.

**Additional file 2.** Nucleotide sequences of primer pairs, product length and specific annealing temperatures applied for the real-time reverse transcriptase polymerase chain reaction (RT- PCR) detection.

**Additional file 3.** Effect of IL-1β (a), TNF-α (b), TGF-β (c) or IL-17 (d) treatment on the mRNA expression of *IL19*, *IL20* and *IL24* of FHs74Int cells.

**Additional file 4.** Effect of IL-1β (a), TNF-α (b), TGF-β (c) or IL-17 (d) treatment on the mRNA expression of *IL20RA*, *IL20RB* and *IL22RA* of FHs74Int cells.

**Additional file 5.** Effect of IL-1β (a), TNF-α (b), TGF-β (c) or IL-17 (d) treatment on the mRNA expression of *IL19*, *IL20* and *IL24* of pdMFs.

**Additional file 6.** Effect of IL-1β (a), TNF-α (b), TGF-β (c) or IL-17 (d) treatment on the mRNA expression of *IL20RA*, *IL20RB* and *IL22RA* of pdMFs.

**Additional file 7.** Effect of IL-1β (a), TNF-α (b), TGF-β (c) or IL-17 (d) treatment on the mRNA expression of *IL19*, *IL20* and *IL24* of PBMCs of children with coeliac disease (CD).

**Additional file 8.** Effect of IL-24 on TGF-β induced ECM deposition of pdMFs. Collagen deposition (a) was investigated by SiriusRed assay (n = 5).

## Data Availability

The datasets used and/or analysed during the current study are available from the corresponding author on reasonable request.

## Abbreviations

α-SMA: α smooth muscle actin
CD: coeliac disease
ECM: extracellular matrix
EGF: epidermal growth factor
FN: fibronectin
FBS: fetal bovine serum
GFD: gluten free diet
IBD: inflammatory bowel disease
IL-: interleukin-
LDH: lactate dehydrogenase
MF: myofibroblasts
PBMC: periferial blood mononuclear cell
PDGF-B: platelet-derived growth factor B
pdMF: primary duodenal myofibroblast
RT: room temperature
TGF-β: transforming growth factor β
TF: traction force
TNF-α: tumor necrosis factor α
tTG: tissue transglutaminase
