# Supplementary material for: Role of IL-24 in the mucosal remodeling of children with coeliac disease
Source: J Transl Med. 2020 Jan 23;18:36. doi: 10.1186/s12967-020-02221-2 (PMC6977354; doi:10.1186/s12967-020-02221-2)
Supplement: Supplementary file 1 — Additional file 1. Clinical characteristics and laboratory parameters of controls and pediatric CD patients involved into duodenal sample collection. [file 12967_2020_2221_MOESM1_ESM.docx]

**Additional file 1. Clinical characteristics and laboratory parameters of controls and pediatric CD patients involved into duodenal sample collection**

|  | control | CD |
| --- | --- | --- |
| Number of patients | 14 | 16 |
| Gender | 10 M / 4 F | 7 M / 9 F |
| Age (mean±SD) | 12.71±4.81 | 6.75±3.38 |
| Lymphocyte number | normal <40% | >40% |
| Anti tTg IgA(U/mL) | <20 | 125-291 |
| Anti tTg IgG (U/mL) | <20 | 6-180 |

Abbreviations: CD: coeliac disease, M: male, F: female, tTg: tissue transglutaminase
