# Supplementary material for: Role of IL-24 in the mucosal remodeling of children with coeliac disease
Source: J Transl Med. 2020 Jan 23;18:36. doi: 10.1186/s12967-020-02221-2 (PMC6977354; doi:10.1186/s12967-020-02221-2)
Supplement: Supplementary file 2 — Additional file 2. Nucleotide sequences of primer pairs, product length and specific annealing temperatures applied for the real-time reverse transcriptase polymerase chain reaction (RT- PCR) detection. [file 12967_2020_2221_MOESM2_ESM.docx]

**Additional file 2. Nucleotide sequences of primer pairs, product length and specific annealing temperatures applied for the real-time reverse transcriptase polymerase chain reaction (RT- PCR) detection.**

| **Gene** | **NCBI ref. seq.** | **Primer pairs** | | **Product length** | **T_a_** |
| --- | --- | --- | --- | --- | --- |
|  |  |  |  |  |  |
| ***ACTA2*** [43] | NM_001141945.2 | F: | 5'- ACT GAG CGT GGC TAT TCC TCC GTT -3' | 111 bp | 58 °C |
|  |  | R: | 5'- GCA GTG GCC ATC TCA TTT TCA -3' |  |  |
| ***ACTB*** [43] | NM_001101.3 | F: | 5'- ACC GAG CGT GGC TAC AGC TTC ACC -3' | 114 bp | 53 °C |
|  |  | R: | 5'- AGC ACC CGT GGC CAT CTC TTT CTC G-3' |  |  |
| ***CDKN1A*** | NM_001220777.1 | F: | 5'- TTG TAC CCT TGT GCC TCG CTC AGG -3' | 126 bp | 60 °C |
|  |  | R: | 5'- ATC AGC CGG CGT TTG GAG TGG TAG -3' |  |  |
| ***COL1A1*** | NM_000088.3 | F: | 5'- CTG CCC CGG CGC CGA AGT C -3' | 96 bp | 60 °C |
|  |  | R: | 5'- CCC TCG ACG CCG GTG GTT TCT TG -3' |  |  |
| ***COL3A1*** | NM_000090.3 | F: | 5'- GTC CCC TGG CTC AAA TGG CTC AC -3' | 113 bp | 56 °C |
|  |  | R: | 5'- GGG GCC CCT TGC TCC TAT TAG TCC -3' |  |  |
| ***FN1*** | NM_212482.2 | F: | 5'- GGC TGC CCA CGA GGA AAT CTG C -3' | 229 bp | 58 °C |
|  |  | R: | 5'- GTG CCC CTC TTC ATG ACG CTT GTG -3' |  |  |
| ***IL1A*** | NM_000575.4 | F: | 5'- TCC TCC ATT GAT CAT CTG TCT CTG -3' | 152 bp | 60 °C |
|  |  | R: | 5'- ACC ACC ATG CTC TCC TTG AA -3' |  |  |
| ***IL1B*** | NM_000576.2 | F: | 5'- CAC GCT CCG GGA CTC ACA G -3' | 160 bp | 56 °C |
|  |  | R: | 5'- GCC CAA GGC CAC AGG TAT TTT -3' |  |  |
| ***IL19*** | NM_153758.2 | F: | 5'- CAC CTT CCC AAA TGT CAC TAT-3' | 95 bp | 51 °C |
|  |  | R: | 5'- ACG CCA GGA GGT TCT TG -3' |  |  |
| ***IL20*** | NM_018724.3 | F: | 5'- CGC CAA TTC CTT TCT TAC CAT CAA -3' | 157 bp | 56 °C |
|  |  | R: | 5'- TTC CCC CAA AGC CTT CAC AAC T -3' |  |  |
| ***IL20RA*** | NM_014432.3 | F: | 5'- CTG CGT ACA CGT GGA GTC CTT-3' | 148 bp | 55 °C |
|  |  | R: | 5'- CAC GGT AAT AGA TAT GGG CAA AAC-3' |  |  |
| ***IL20RB*** | NM_144717.3 | F: | 5'- GGT GCC GAG GAA CAT GTC AAA AT -3' | 188 bp | 58 °C |
|  |  | R: | 5'- AGG GCC AGT ACC AGG GGA ATG-3' |  |  |
| ***IL22RA*** | NM_021258.3 | F: | 5'- TGC CCC CTA CAT GTG CCG AGT GAA -3' | 145 bp | 50 °C |
|  |  | R: | 5'- AGG TGC AGG CGG CTT GGT GAC ATA -3' |  |  |
| ***IL6*** | NM_000600.4 | F: | 5'- AAA GAT GGC TGA AAA AGA TGG AT -3' | 146 bp | 54 °C |
|  |  | R: | 5'- CTC TGG CTT GTT CCT CAC TAC TCT -3' |  |  |
| ***KI67*** | NM_002417.4 | F: | 5'- CCC CTA CGG ATT ATA CTC AAC TTA -3' | 217 bp | 54 °C |
|  |  | R: | 5'- TGT AAT ATT GCC TCC TGC TCA T -3' |  |  |
| ***IL24*** | NM_006850.3 | F: | 5'- AGG CGG TTT CTG CTA TTC C -3' | 55 bp | 48 °C |
|  |  | R: | 5'- GAG CTG CTT CTA CGT CCA ACT -3' |  |  |
| ***PCNA*** | NM_002592.2 | F: | 5'- GCG GTC TGA GGG CTT CGA CAC CTA -3' | 134 bp | 60 °C |
|  |  | R: | 5'- CCG CGT TAT CTT CGG CCC TTA GTG -3' |  |  |
| ***RPLP0*** | NM_001002.3 | F: | 5'- GGG GGA ATG TGG GCT TTG TGT T -3' | 206 bp | 59 °C |
|  |  | R: | 5'- GGT GCC CCT GGA GAT TTT AGT GGT -3' |  |  |
| ***SNAI1*** | NM_005985.3 | F: | 5'- GCC ATG TCC GGA CCC ACA CTG -3' | 152 bp | 60 °C |
|  |  | R: | 5'- TCG GGA GAA GGT CCG AGC ACA C -3' |  |  |
| ***SNAI2*** | NM_003068.4 | F: | 5'- CGC GCT CCT TCC TGG TCA -3' | 159 bp | 60 °C |
|  |  | R: | 5'- GGC TGT ATG CTC CTG AGC TG -3' |  |  |
| ***TP53*** | NM_001126118.1 | F: | 5'- TGG TCT GGC CCC TCC TCA GCA TCT -3' | 114 bp | 60 °C |
|  |  | R: | 5'- TCA GGC GGC TCA TAG GGC ACC AC -3' |  |  |
| ***TNF*** | NM_000594.4 | F: | 5'- GAG GCG CTC CCC AAG AAG ACA -3' | 182 bp | 60 °C |
|  |  | R: | 5'- TGG GCC AGA GGG CTG ATT AGA G -3' |  |  |
| ***VIM*** | NM_003380.4 | F: | 5'- GAG GCT GCC AAC CGG AAC AAT GAC -3' | 203 bp | 60 °C |
|  |  | R: | 5'- TCC TGC AGG CGG CCA ATA GTG TCT -3' |  |  |

Abbreviations: ref. seq.: reference sequence; F: forward; R: reverse; bp: base pair; T_a_: annealing temperature
