# Supplementary material for: Role of IL-24 in the mucosal remodeling of children with coeliac disease
Source: J Transl Med. 2020 Jan 23;18:36. doi: 10.1186/s12967-020-02221-2 (PMC6977354; doi:10.1186/s12967-020-02221-2)
Supplement: Supplementary file 3 — Additional file 3. Effect of IL-1β (a), TNF-α (b), TGF-β (c) or IL-17 (d) treatment on the mRNA expression of IL19, IL20 and IL24 of FHs74Int cells. [file 12967_2020_2221_MOESM3_ESM.docx]

**Additional file 3.**

Additional file 3.jpg


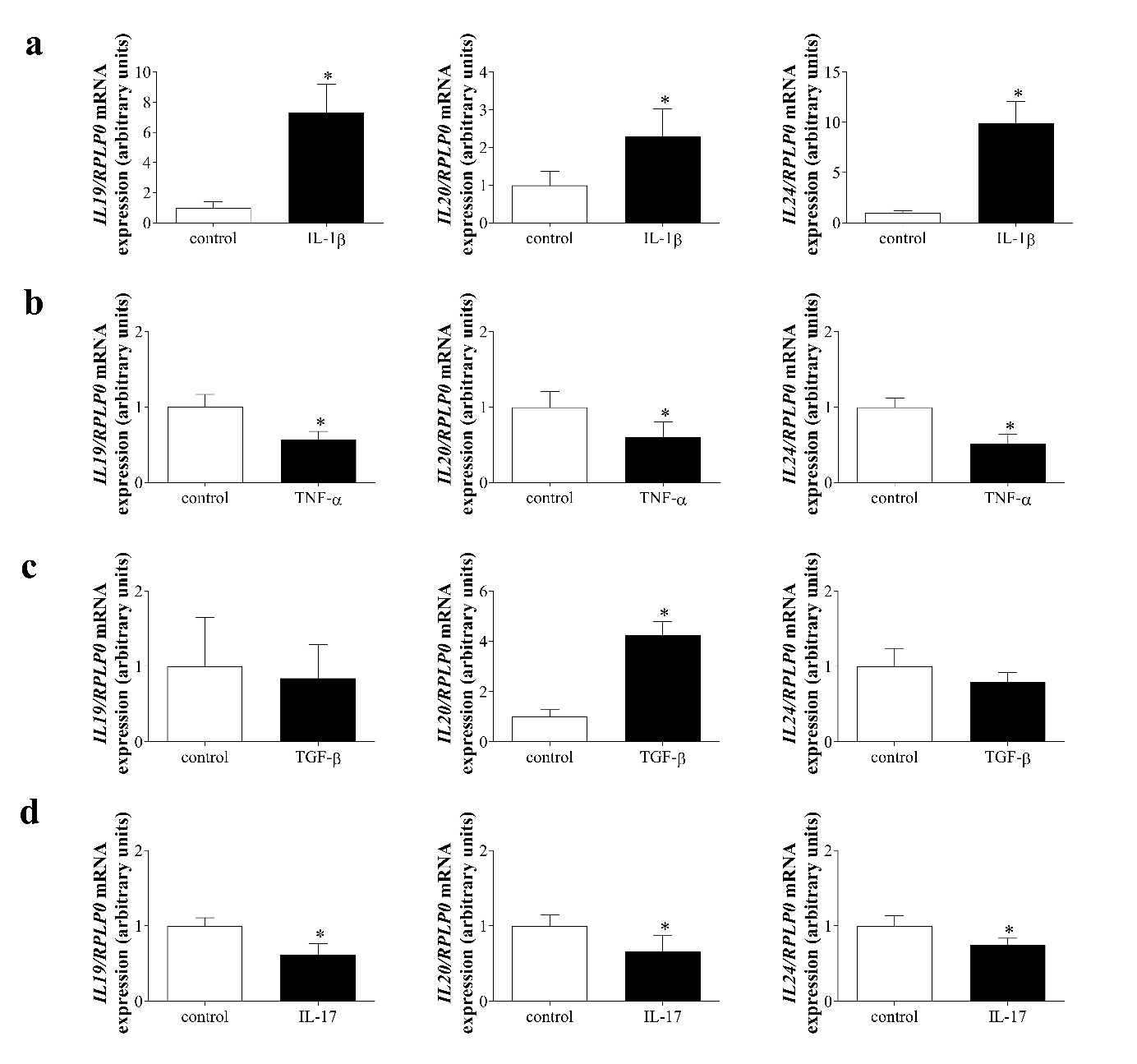


**Additional file 3. Effect of IL-1β (a), TNF-α (b), TGF-β (c) or IL-17 (d) treatment on the mRNA expression of *IL19*, *IL20* and *IL24* of FHs74Int cells.** The mRNA expression of *IL19*, *IL20* and *IL24* was measured by real-time RT-PCR (n=6). Relative mRNA expression was determined by comparison with *RPLP0* as internal control. Results are presented as mean+SD. *p<0.05 vs. control (Mann-Whitney U-test).
