# Supplementary material for: Role of IL-24 in the mucosal remodeling of children with coeliac disease
Source: J Transl Med. 2020 Jan 23;18:36. doi: 10.1186/s12967-020-02221-2 (PMC6977354; doi:10.1186/s12967-020-02221-2)
Supplement: Supplementary file 4 — Additional file 4. Effect of IL-1β (a), TNF-α (b), TGF-β (c) or IL-17 (d) treatment on the mRNA expression of IL20RA, IL20RB and IL22RA of FHs74Int cells. [file 12967_2020_2221_MOESM4_ESM.docx]

**Additional file 4.**

Additional file 4.jpg


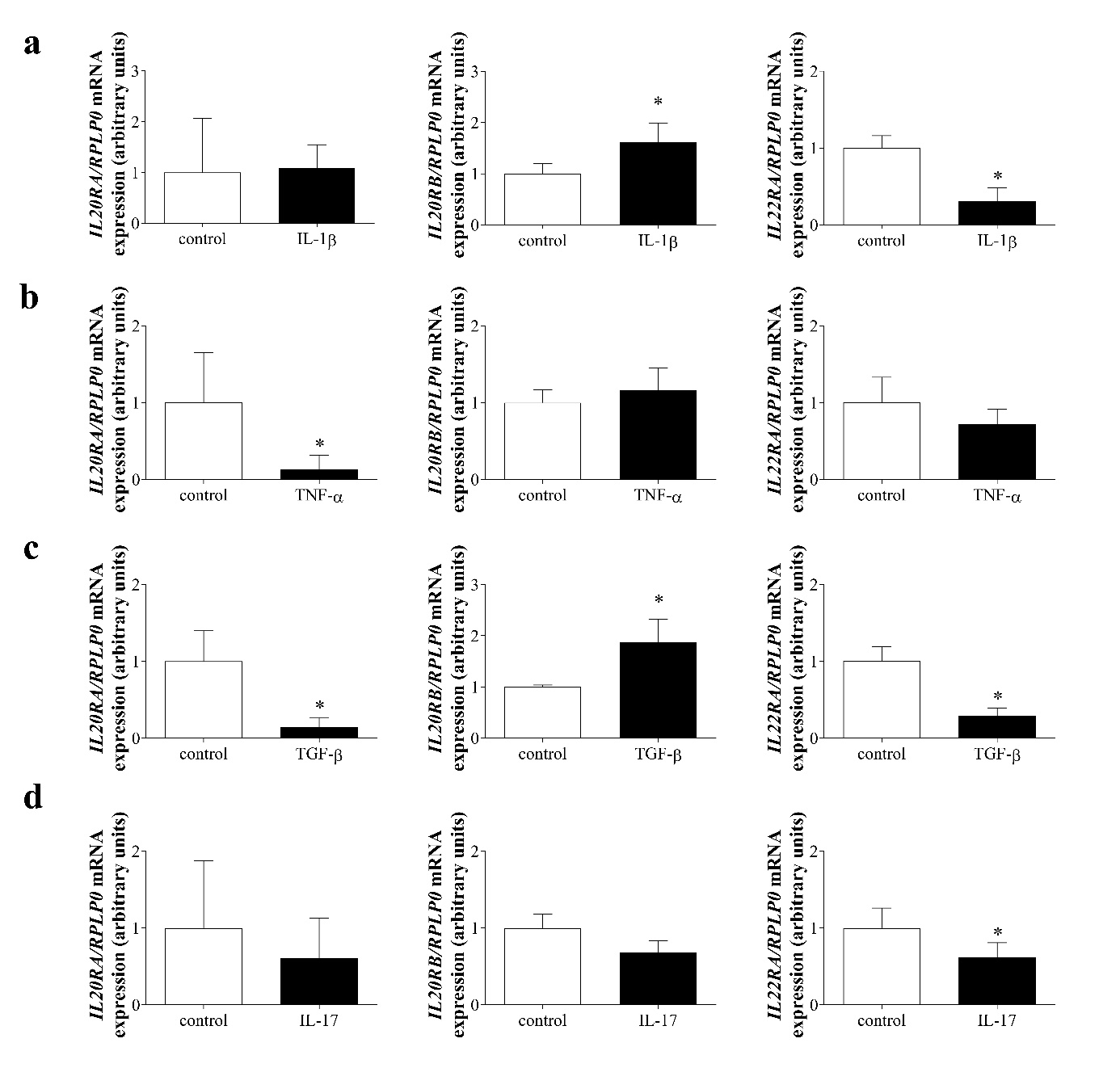


**Additional file 4. Effect of IL-1β (a), TNF-α (b), TGF-β (c) or IL-17 (d) treatment on the mRNA expression of *IL20RA*, *IL20RB* and *IL22RA* of FHs74Int cells.** The mRNA expression of *IL-20RA*, *IL20RB* and *IL22RA* was measured by real-time RT-PCR (n=6). Relative mRNA expression was determined by comparison with *RPLP0* as internal control. Results are presented as mean+SD. *p<0.05 vs. control (Mann-Whitney U-test).
