# Supplementary material for: Role of IL-24 in the mucosal remodeling of children with coeliac disease
Source: J Transl Med. 2020 Jan 23;18:36. doi: 10.1186/s12967-020-02221-2 (PMC6977354; doi:10.1186/s12967-020-02221-2)
Supplement: Supplementary file 8 — Additional file 8. Effect of IL-24 on TGF-β induced ECM deposition of pdMFs. Collagen deposition (a) was investigated by SiriusRed assay (n = 5). [file 12967_2020_2221_MOESM8_ESM.docx]

**Additional file 8.**

Additional file 8.jpg


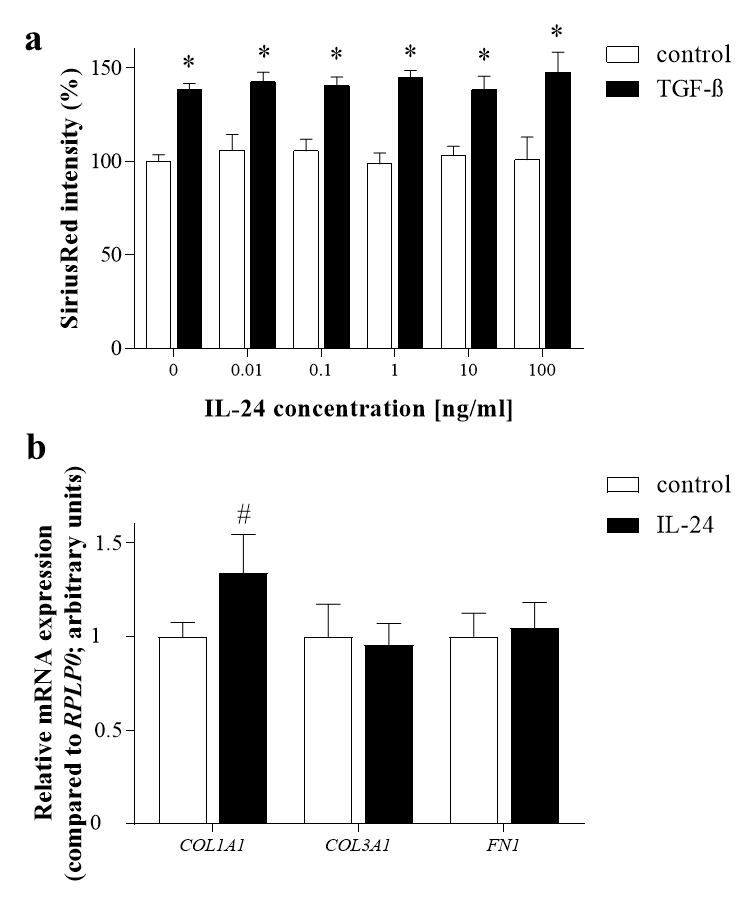


**Additional file 8. Effect of IL-24 on TGF-ß induced ECM deposition of pdMFs.** Collagen deposition (a) was investigated by SiriusRed assay (n=5). Results are presented as the percentage of the untreated group (0 ng/mL IL-24 control). The mRNA expression of *COL1A1*, *COL3A1* and *FN1* (b) was measured by real-time RT-PCR (n=5). Relative mRNA expression was determined by comparison with *RPLP0* as internal control. Results are presented as mean+SD *p<0.05 vs. *control* (multiple t-test), #p<0.05 vs. *control* (Mann-Whitney U-test).
